# A meta-analysis shows that seaweeds surpass plants, setting life-on-Earth’s limit for biomass packing

**DOI:** 10.1186/s12898-019-0218-z

**Published:** 2019-01-31

**Authors:** Joel C. Creed, Vasco M. N. C. S. Vieira, Trevor A. Norton, Debora Caetano

**Affiliations:** 1 Departamento de Ecologia, Instituto de Biologia Roberto Alcântara Gomes, Universidade do Estado do Rio de Janeiro, Rua São Francisco Xavier 524, Rio de Janeiro, RJ 20550-900 Brazil; 2 MARETEC, Instituto Superior Técnico, Universidade Técnica de Lisboa, Av. Rovisco Pais, 1049-001 Lisbon, Portugal; 3 Port Erin Marine Laboratory, University of Liverpool, Port Erin, Isle of Man IM9 6JA UK

**Keywords:** Algae, Biomass, Density, Meta-analysis, Self-thinning

## Abstract

**Background:**

As plants, algae and some sessile invertebrates may grow in nearly monospecific assemblies, their collective biomass increases and if they compete hard enough some die, freeing up space. The concurrent increase in biomass and decrease in density is called self-thinning, and its trajectory over time or maximum values represent a boundary condition. For a single stand developing over time the boundary defines the carrying capacity of the environment but the most extreme trajectories emulate the efficiency of species in packing biomass into space.

**Results:**

Here we present a meta-analysis of compiled data on biomass and density from 56 studies of 42 species of seaweeds from 8 orders within 3 phyla scattered through the world’s oceans. Our analysis shows that, with respect to biomass, seaweeds are the most efficient space occupiers on Earth because they transgress previously fixed limits derived from land plants. This is probably because seaweeds are not limited by water and do not need structures for its transport or for transpiration; they photosynthesise and uptake nutrients over their entire surface; they are attached to the substrate by holdfasts that are small proportional to their volume or weight compared to roots; water provides them better support, reducing the need for tissues for rigidity. We also identified a biomass concentration common to plants and seaweeds which represents the threshold that no life on the planet can pass. Using each stand’s distance to the biomass–density boundary, we determined that within the seaweeds the efficiency of space occupation differed amongst taxonomic and functional groups as well as with clonality and latitude.

**Conclusions:**

Algae occupy space more efficiently than plants, most likely because the watery environment facilitates the physical processes and integration of space occupation. The distance-to-the-boundary proves a good metric to discriminate among groups and may be useful for comparison of the most efficient biomass producing systems, or for the identification of systems impacted by pollution.

**Electronic supplementary material:**

The online version of this article (10.1186/s12898-019-0218-z) contains supplementary material, which is available to authorized users.

## Background

Self-thinning is the decrease in density (death) within an even-aged monospecific plant or algae stand and is driven by biomass increment (growth). It occurs because, above a certain threshold, intraspecific competition for finite resources induces mortality, which releases space and resources for the survivors to use. The generality of the relationship between density (D) and mean plant mass (w) has been recognised since the 1950s in populations from a range of species [1–4]. This relation is given by the self-thinning slope, *w *= *k*_*w*_*D*^−3/2^ or equivalently log_10_*w *= log_10_*k*_*w*_− 1.5log_10_*D*, and the generality of the relationship previously termed “Yoda’s law”, “− 3/2 power law”, “self-thinning rule”, “power law of self thinning” and “− 3/2 thinning law” was considered “the only generalization worthy of the name law in plant ecology” [5]. It has also been applied to some mixed species stands [6] and marine animals [7, 8].

Another line of evidence is derived regressions across species of different sized plants (e.g. [2, 9, 10]), which generally form a − 3/2 gradient band. The “law” attracted plant ecologists’ attention and an improved relationship between stand biomass per unit area (*B*) and density [10]: *B *= *k*_*B*_*D*^−1/2^ or equivalently log_10_*B *= log_10_*k*_*B*_ − 0.5log_10_*D* was established, which resolved the previous problem of auto-correlation. This was because the *w*–*D* relationship required the number of individuals to estimate the quantities on both sides of the equation. It also eliminated another problem in that mean biomass could increase without actual growth simply due to the fact smaller individuals died [4, 11]. Subsequently, further there were advances to better assess the biomass–density relationship, such as better data quality selection and choice of regression methods [4, 12–15]. There was also significant debate and controversy as to what the self-thinning “law” represented and if in fact a law really existed. Resulting from this debate and improvements the biomass–density relations may be divided into three different features:

- The dynamic thinning line—a straight line that is approached and followed by the path of an individual crowded stand over time [12, 13] on a log–log plot. This dynamic thinning line’s gradient and intercept are customarily related to the allometry of a plant species [16] as well as limitations, such as nutrient availability or temperature [17–19]; flatter slopes, with lower intercepts reflect reduced carrying capacities;
- The species boundary line—the upper boundary of possible biomass–density combinations for any given species of plant or algae. Ideally this line is fit to the most extreme of hundreds of stands [12, 13] and theoretically the y-intercept of a species provides information about its capacity to pack biomass;
- The interspecific biomass–density relationship—a static upper limit characterizing the maximum biomass–density boundary for all plants and algae. Weller [20] analysed plant data setting the boundary at log_10_*B* = 3.91 − 0.33log_10_*D*. Scrosati [21] re-analysis of Weller’s data set the plant boundary at log_10_*B* = 4.87 − 0.33log_10_*D*.

Regarding seaweeds, Schiel and Choat [22] examined two species which possess quite different forms and life histories. Their data demonstrated that the dry weight increased with increasing frond density, concluding that the − 3/2 thinning law was unlikely to apply to seaweeds. Schiel and Choat’s [22] data did not trace a time series so only the species boundary line should have been fit, for which hundreds of points are ideal. Shortly after, Cousens and Hutchings [23] considered self-thinning in six brown seaweeds, which they compared to an interspecific boundary line (using the log_10_*w*) with a 4.3 intercept, then considered to be the highest known for land plants [2]. The points fell on or below the boundary line so Cousens and Hutchings [23] concluded that seaweeds did not violate the law, and pointed out that Schiel and Choat’s [22] data did not contravene their ‘all species boundary line’. Since then there have been few studies of self-thinning in seaweeds, several of which (Robertson [24], Cheshire and Hallam [25], Russell [26] and Martínez and Santelices [27]) have rejected the findings by Cousens and Hutchings’ [23]. Those studies did not use the recommendations of Weller [4] for selecting data points, fitting slopes and analysing the data that are essential for robust interpretations [28, 29]. Other studies of seaweeds (Flores Moya et al. [28], Creed [30], Creed et al. [31], and Arenas and Fernández [32]) applied Weller’s [4] ‘best practices’ to estimate the log_10_*B* − log_10_*D* relation and concluded that the biomass–density slopes were not significantly different from − 0.5.

Humans use biomass, both from terrestrial [33] and aquatic [34, 35] autotrophs in numerous ways for their well being. Autotrophic biomass is also at the base of most food chains, so, understanding which sources provide this service most efficiently is not trivial. As aquatic organisms seaweeds have traits that are inherently different from terrestrial plants and we hypothesise that these traits may make them pack biomass differently and better. There are now sufficient data on biomass and density in seaweeds that a synthesis and investigation of this hypothesis is possible. For this we analyse compiled data on biomass and density from 56 studies about 42 species of seaweeds from 8 orders within 3 phyla scattered through the world’s oceans (Additional file 1). We use a subset of these data to estimate the seaweed’s interspecific boundary line (IBL) and determine whether or not seaweeds and terrestrial plants share the same IBL.

The classical models predict a boundary line based on (i) the volume available to each plant, in its turn dependent on how tall the plant can grow, (ii) the plant shape and its ability to occupy the available volume, and (iii) the biomass packed per unit of volume effectively used. In plants as well as in seaweeds, these characteristics are more likely to change according to taxonomic group, functional form and environment. Consequently, we use each stand’s position relative to the IBL as an index of the efficiency of space occupation to test whether seaweeds differentiate in their efficiencies according to taxa, functional forms and latitude.

The intraspecific dynamic biomass–density relationship (self-thinning) often does not apply to clonal plants and seaweeds. Because several fronds may arise from the same genetic individual, being physiologically connected through roots or holdfast, these may not compete but instead cooperate, sharing acquired resources [36]. Consequently, an increase in biomass may arise from an increase in frond size and/or in density. Hutchings [36] identified several non-thinning biomass–density dynamics typical of clonal plants. Later, Westoby [37] and de Kroon and Kalliola [38] determined that clonal plants may (or not) self-thin depending on the specificities of their life-histories and their interactions with external biotic and abiotic factors. The occurrence of self-thinning is also variable in clonal algae and seems to depend on their specific life-history, morphological characteristics and habitat [21, 39–42]. Although not necessarily self-thinning, terrestrial clonal plants were nevertheless demonstrated to be limited by an IBL [36]. We use each stand’s position relative to the IBL to determine whether, irrespective of dynamically self-thinning or not, clonal algae show the same efficiency of space occupation of non-clonal algae.

Synthesizing the aim of the present study (as detailed above) with regard to the efficiency of biomass packing, using data compiled from studies in the literature and some personal observations we propose to test whether: (i) seaweeds are better than terrestrial plants, (ii) seaweeds with simple forms are better than complex ones, (iii) clonal seaweeds are different from non-clonal ones, and (iv) latitude affects seaweed efficiency.

## Methods

### Collecting and pre-processing the data

The data collection started 25 years ago and was carried out until 2017. In later years we used the Google search engine as well as the search engines in the webpages of the cited publications (no date limit). The English language search keywords included ‘biomass’, ‘density’, ‘self-thinning’, ‘boundary’, ‘seaweed’, ‘alga’ and its derivatives, and the scientific denominations of taxa. We also searched the reference lists in the works that we cite and the *curriculum vitae* of their authors for further studies or data. The original data were requested from authors. When this proved impossible or data were not forthcoming, data were taken from publications (Additional file 1). In these cases the data was either provided in tables or we magnified the respective figures to the most adequate scale and estimated values. In addition, unpublished data of dry weight and frond density collected by Dr. Joel Creed in the Isle of Man, UK (1989–1993) and at Rio de Janeiro, Brazil (1999) were included. The full meta-analysis data comprised 1856 observations distributed among 42 species (using current taxonomic status from AlgaeBase—http://www.algaebase.org).

The *Macrocystis pyrifera* wet weights taken from Van Tussenbroek [43] were converted to dry weight applying a 0.11 coefficient [44]. The *Macrocystis pyrifera* dry weights [43] was corrected by addition of the dry weight of the holdfasts estimated from Van Tussenbroek [43, 44] and 2% of the estimated plant dry weight to compensate for the missing sporophyll [45]. The *Iridaea cordata* [46] and the *Phyllophora antartica* [47] wet weights were converted into dry weights by applying a 0.126 dry:wet ratio averaged from 14 estimates reported by Goreau and Trench [48].

The use of *mean* plant dry biomass has two drawbacks [4, 14, 29]: firstly, *mean* plant biomass may increase solely because small plants die, not because of growth of plants within the population; secondly, because *mean* plant biomass is calculated from stand biomass (i.e. mean plant mass = stand biomass/density), and thus can easily lead to autocorrelation. Therefore, the data were pre-processed so values reported as mean frond or plant weight were converted into stand biomass.

### Screening the data

Weller [4] questioned the ecological validity of using data of non-thinning stands for the estimation of the IBL. We add a numerical argument for a careful selection of the data from such stands: these are randomly scattered below the IBL, leading their distribution to approximate a circle. In these cases the slopes estimated by ordinary least squares (OLS) tend to 0, those estimated by reduced major axis (RMA) tend to 1, and those estimated by principal components analysis (PCA) become erratic [49–52]. We estimated the boundary line using quantile regression (QR). Although this method is robust to outliers, it becomes as prone to bias as do the OLS, RMA and PCA if the majority of observations are from non-thinning stands ‘inlying’ the self-thinning trend. On the other hand, although clonal algae did not self-thin, several of their stands were nevertheless sufficiently close to the boundary to aid in its numerical estimation. Hutchings [36] had already demonstrated that clonal autotrophs, even not self-thinning, are nevertheless limited by the same boundary of non-clonal autotrophs. These two aspects led to us carefully discriminating stands valid for the IBL estimation, which resulted in the selection of 138 observations distributed among the 9 species from the Chlorophyta, Gelidiales, Laminariales and Fucales for which most data were available. These data are provided as Additional file 2.

### Determining the IBL

The IBL line-fit was performed applying the quantile regression (QR) method with a 99% threshold. This method estimates the coefficients that approximate the conditional median or quantiles of the response variable given the distribution of the predictor. Its traditional type I regression algorithm (QR1)—i.e., minimizing the vertical residuals by assuming that only the response variable is measured with error—was the simpler among those that proved reliable estimators of the interspecific boundary line for terrestrial plants, and is fairly insensitive to outliers when compared to the OLS [15].

1 $$B_{\tau } = \mathop {\text{argmin}}\limits_{B} \left[ {\left( {1 - \tau } \right)\mathop \sum \limits_{{y_{i} < \beta X_{i} }} \left( {BX_{i} - y_{i} } \right) + \tau \mathop \sum \limits_{{y_{i} \ge \beta X_{i} }} \left( {y_{i} - BX_{i} } \right)} \right]$$

The regression coefficients *Β*_*τ*_ = {*β*_*0*_,*β*_*1*_} for the linear regression *y *= *β*_*0*_+ *β*_*1*_× *x* relative to the τ quantile (in this case *τ *= 0.99) were more efficiently estimated using matrix algebra. This required defining the (*n *× 2) matrix **X** with numbers ‘1’ in the first column and the observed *x* in the second, and where *n* is the number of observations. Then, *Β* was iteratively estimated from Eq. 1 applying an efficient linear programming strategy: an initial *Β* estimate provided by OLS approximated reasonably well the *Β*_*0.5*_ (i.e., for the median). This first guess was close enough from the *Β*_*0.99*_ to use as starting point. The right hand side of Eq. 1 (i.e., the objective function f) was estimated. Then, small proportional increments *δ* were applied to *β* at the time (i.e., _new_*β*_*i*_ = (1 + *δ*)*β*_*i*_) and the objective function f re-estimated. Its evolution in both directions [i.e., f(*δ*) and f(− *δ*)] was tracked. The increments minimizing f were selected and applied to *Β*_*τ*_, simultaneously. This process was applied iteratively until convergence. Reliable estimates always took more than 2 iterations and less than 20 to converge to the 4th decimal. The rate of convergence was determined by the magnitude of *δ* relative to *n*, in a directly proportional relation (i.e., *n*/*δ *≈ C). For example, this search algorithm optimized its convergence applied to the *L. digitata* data selected from Creed et al. [31] (*n *= 21) taking 14 iterations with *δ *= 0.002; applied to the IBL selected data (*n *= 138) taking 9 iterations with *δ *= 0.01; and applied to the full data (*n *= 1856) taking 10 iterations with *δ *= 0.15. Frequently, the convergence did not lead to a stationary point but to small oscillations around a central tendency causing the search algorithm to proceed endlessly. In these cases we introduced (i) a small algorithm stopping the search when such oscillations were identified and estimated their mean *Β*_*τ*_, and (ii) a threshold amount of iterations (t_max_ = 20).

Type I regression has been demonstrated inadequate for line-fitting of data lacking a hierarchical structure (i.e., predictor-response) and/or where both variables have been measured with errors of approximate magnitude, as is the case of biomass–density relations [4, 14, 29]. Hence, the type I quantile regression (QR1) was adapted to a type II regression (QR2). Minimizing the residuals perpendicularly to the regression line is straightforward and was done with the objective function f being adapted into two nested equations (Eqs. 2a and 2b). The angle *θ* between the provisional oblique residuals and the y axis was estimated. From the basic geometry of rotating orthogonal axes results that this is the same angle between the provisional regression line and the x axis (Eq. 2a). The oblique residuals given *θ* were estimated (Eq. 2b). Given the new objective function f, the search algorithm was applied to QR2 similarly to QR1. Both algorithms performed similarly with respect to convergence rate and accuracy.

2a $$\theta = {\text{arctg}}\left| {\beta_{1} } \right|,\quad \quad \forall y_{i} < BX_{i}$$

2b $$B_{\tau } = \mathop {\text{argmin}}\limits_{B} \left[ {\left( {1 - \tau } \right)\mathop \sum \limits_{{y_{i} < \beta X_{i} }} \left| {BX_{i} - y_{i} } \right| + \tau \mathop \sum \limits_{{y_{i} \ge \beta X_{i} }} \left| {y_{i} - BX_{i} } \right|} \right]{ \cos }\theta$$

The Matlab scripts implementing these methods and its tutorial are provided as Additional file 3.

### Estimating the perpendicular distances

**Fig. 1:**
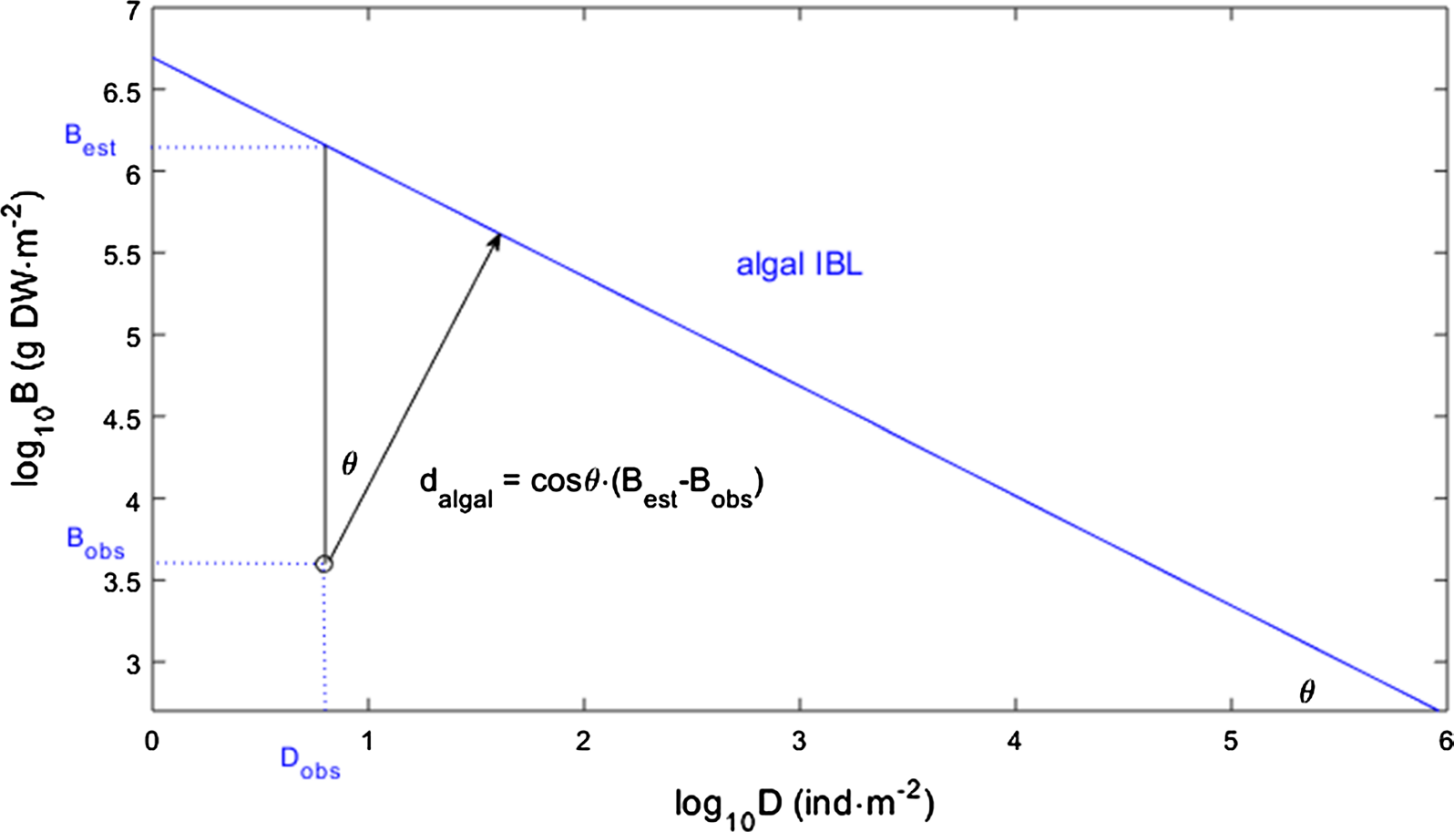
The perpendicular distance to the IBL (d_algal_). This is estimated from the observed (obs) and estimated (est) biomass (B) and density (D), and the algal IBL

In order to estimate the perpendicular distance from each stand to the macroalgal IBL (d_algal_—see Fig. 1) it is necessary to establish the linear coefficients of the boundary line. The IBL equation is given by log_10_*B* = *β*_0_ + *β*_1_log_10_*D* so the coefficients for the IBL for algae were *β*_0_ = 6.694 and *β*_1_ = − 0.67. We based our estimate on the geometry of orthogonal axis rotation, the angle *θ* between the d_algal_ vector (which is perpendicular to the IBL and therefore oblique to the log_10_*B*-to-log_10_*D* orthogonal plane) as well as the log_10_*B* vertical axis, as the same angle between the algal IBL and the log_10_*D* horizontal axis. Hence, *θ* = arct*g*(|*β*_1_|), which for algae is *θ* = 0.59. In order to calculate perpendicular distance to the IBL we used the cosine of *θ* which for algae was cos*θ* = 0.83 multiplied by the log_10_*B* vertical distance: d_algal_ = (log_10_*Ḃ*–log_10_*B*)·cosθ. These vertical distances required the use of observed log_10_B and the estimation of log_10_*Ḃ* = *β*_0_ + *β*_1_log_10_*D*.

### Discriminating among algal groups

While discriminating among groups, to prevent bias from studies having largely different numbers of observations, from each of the 55 studies providing useful data were selected the three lowest distances (i.e., highest efficiencies). Hence, all studies were equally weighted. The observations/distances were grouped and compared according to taxa, functional group, clonality and latitude:

- The taxa used were Chlorophyta, Rhodophyta (Gelidiales, Gigartinales and Bonnemaisonniales) and Phaeophyta (Laminariales, Fucales, Ectocarpales, Desmasrestiales and Tilopteridales).
- The functional groups were classified according to Steneck and Dethier [53] by the seaweeds morphological characteristics and ranked in descending order according to productivity as Foliose Algae (3), Corticated Foliose Algae (3.5), Corticated Macrophytes (4) and Leathery Macrophytes (5). The functional groups ranked 1, 2, 6 and 7 were not represented in the meta-analysis.
- The residual effect of latitude on the distance from the IBL was estimated by filtering out the dominant effects of the taxonomical order. The effect of functional group was not explicitly filtered-out because it already nested the factor ‘order’. Thus the negative adjusted distances represented increasing proximity to the boundary and thus a more efficient occupation of space, whereas the positive adjusted distances represented departure from the boundary and thus a less efficient occupation of space.

## Results

**Fig. 2:**
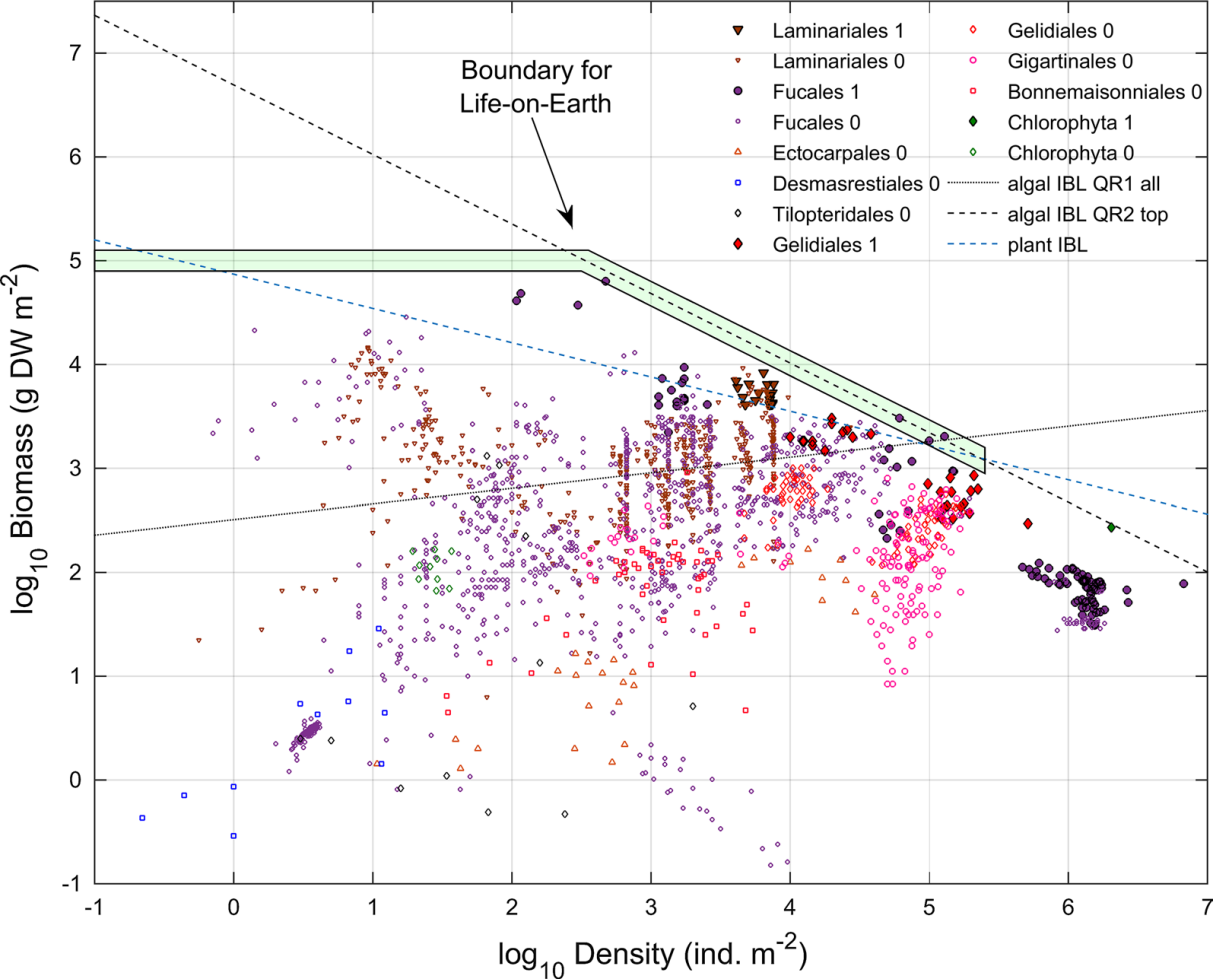
The biomass and density of seaweeds and plants on Earth. The plant interspecific boundary line (plant IBL) was postulated by Weller [20] and Scrosati [21]. The seaweed’s IBL was estimated by model I (QR1) and model II (QR2) quantile regression on all the algal data (all) or to the selected algal data (top). Observations selected (1) or not selected (0)

An accurate estimate of the algal IBL required careful data selection as the use of all available observations, with biomass and density correlated at *r* = 0.036, led the quantile regression to estimate an aberrant IBL (Fig. 2). Among the stands bringing noise to this estimation, there were many conspicuously self-thinning and still well below the true IBL (Fig. 2). These stands below the IBL must be limited by other environmental factors rather than by intrinsic packing into space and their inclusion compromised the accuracy of the IBL estimate. On the other hand, when selecting exclusively the observations closer to the top-right corner of the log_10_*B*–log_10_*D* plot, the biomass and density correlated at *r* = 0.92. Permutation tests with 1000 iterations demonstrated that this correlation was significant (*p* < 0.001). This cluster of observations included stands under clear biomass–density limitation although not conspicuously following a self-thinning time trajectory. The type II quantile regression (QR2) applied to this cluster estimated an algal IBL that fitted the extreme observations remarkably well (Fig. 2), yielding a slope *β*_1_ = − 0.67 (reporting to *k*_*B*_) and an intercept *β*_0_ = 6.694. The fact that this cluster comprised four species from Fucales (Phaeophyta), one from Laminariales (Phaeophyta), two from Gelidiales (Rhodophyta) and one from Chlorophyta provided robustness about the generality of this IBL estimate for algae.

**Fig. 3:**
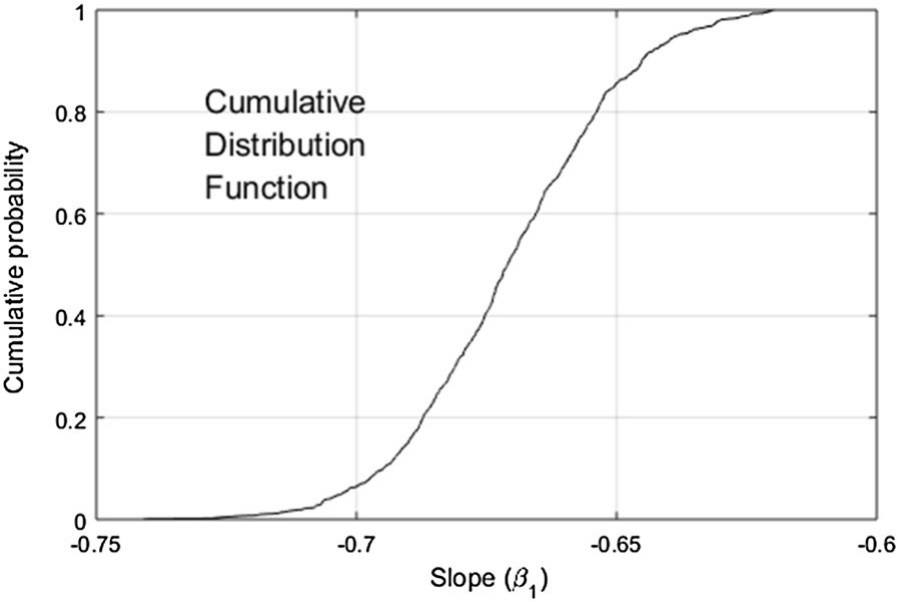
Cumulative distribution function of the slope (β_1_) of the bootstrapped algal interspecific boundary line (IBL)

The algal and plant boundaries were clearly distinct. Bootstrap tests with 1000 iterations demonstrated that the seaweed observations could not yield the − 0.5 slope postulated for terrestrial plants in earlier studies about self-thinning, or the − 0.33 slope later postulated by Weller [20] and Scrosati [21] (*p* < 0.001, Fig. 3). Terrestrial plants clearly differed from seaweeds when the plant IBL (log_10_*B* = 4.87 − 0.33log_10_*D*) used by Scrosati [21] was plotted together with the algal data and IBL (Fig. 2). To put this into perspective, the re-analysis performed by Scrosati [21] was based on the data of Weller [20] including stands of some of the biggest and most densely forested trees on the planet such as *Sequoia sempervirens*, *Tsuga heterophylla*, *Psuedotsuga menziesii*, six *Eucaliptus* spp. and 15 *Pinus* spp. These stands usually varied between 1 and 100 kg dry mass m^−2^, and only two slightly exceeded the 100 kg dry mass m^−2^ threshold. These tree stands occurred at roughly 0.1 stems m^−2^ density [20]. At this same density the algal IBL predicts an expected biomass of ≈ 23 tonnes dry mass m^−2^.

**Fig. 4:**
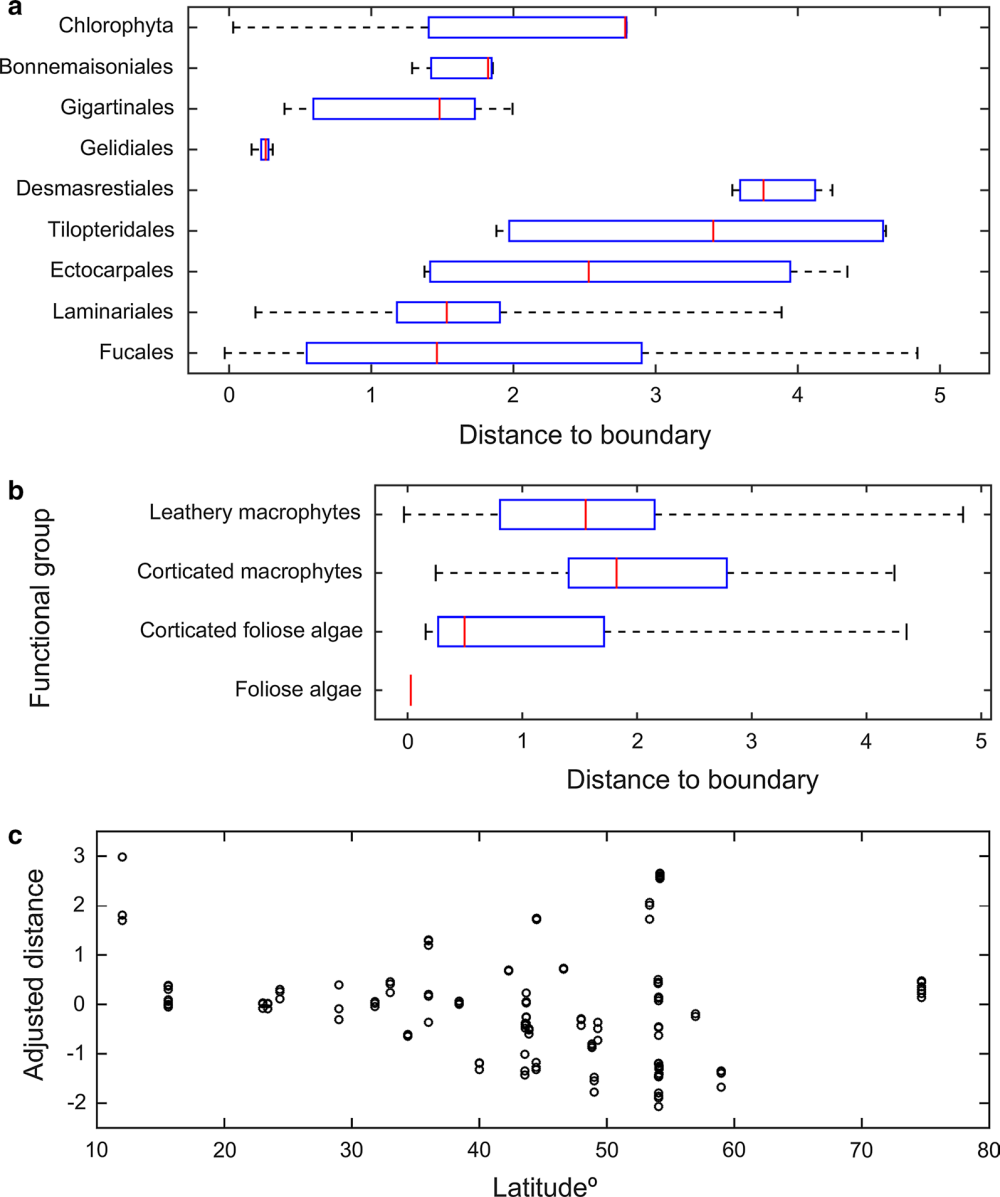
Effects of taxonomic group, functional group and latitude on the algal efficiency of space occupation. Efficiency estimated as the orthogonal distance to the biomass–density boundary. The line bars indicate 0% and 100% of the observations, the boxes indicate the 25% and 75% quartiles and the red line is the median. The adjusted distance was estimated as the raw distance subtracted by the mean distance estimated for the jth taxa

The efficiency of space occupation in each observation of an algal stand was estimated by the minimum perpendicular distance to the algal IBL (an oblique vector) and to the proposed log_10_*B* = 5 (100 kg dry mass m^−2^) general boundary (a vertical vector), with lower distances representing higher efficiencies. Grouping these distances by taxa demonstrated how the orders within each phylum can be well separated according to their space occupation efficiency (Fig. 4a). Grouping the distances by functional group demonstrated that the morphologically simpler (and more productive) algae [53] occupy space more efficiently (Fig. 4b): The bulk of the observations of corticated foliose algal stands were closer to the biomass–density boundary than the bulk of the observations of corticated and leathery macrophytes. Hence, simpler body construction, better space occupation and productivity were all well correlated.

Two clear trends with increasing geographical distance from the equator were revealed (Fig. 4c): (i) efficiency was increasingly variable, and (ii) stands could get increasingly closer to the biomass–density boundary (i.e. more efficiently occupying space). However, it is not clear whether this trend related to the environment or to the fact that most studies were performed by developed countries in their own territories (typically at higher latitudes), whereas studies carried out in developing countries (typically at lower latitudes) were under-represented (see Additional file 1).

**Fig. 5:**
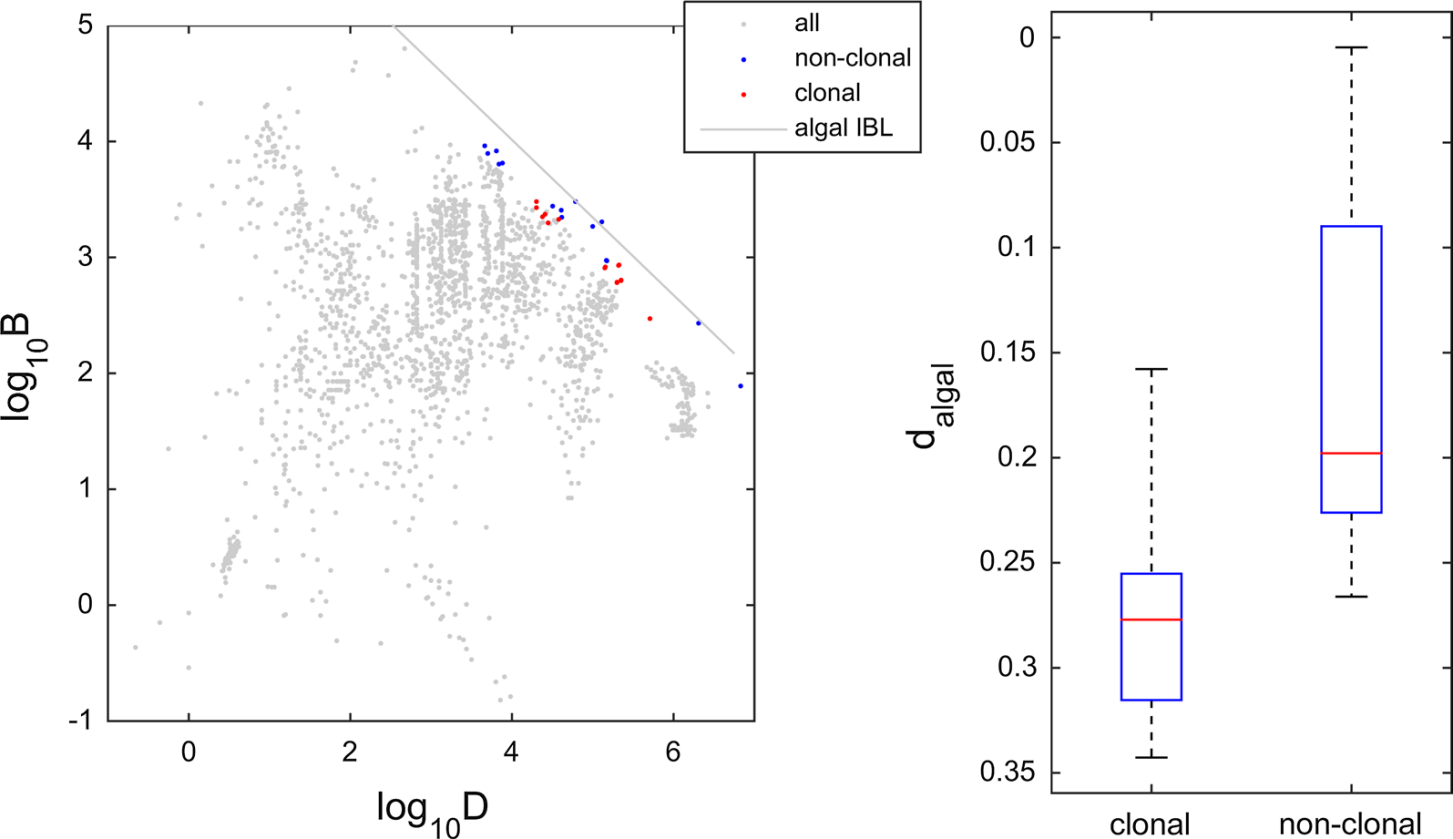
Perpendicular distance to the algal IBL (d_algal_) of clonal and non-clonal stands. For an honest comparison, only the closer 15 stands of each type were used. Box and whiskers correspond to the quartiles

The algal meta-analysis comprised 431 stands of species well known to be clonal and 1182 stands of species well known to be non-clonal. There were still some stands whose category we did not know (see Additional file 1). However, to compare the efficiency of space occupation of clonal and non-clonal algal stands we chose only those stands that had been previously selected to estimate the algal IBL i.e., the ones used in the quantile regression (see Additional file 1). Clonal algae were represented by 2 species, and in both cases the authors of the respective studies measured ramets. If genets had been measured instead of ramets, the stand biomass would stay the same but the stand density would decrease, placing these clonal algae stands further below the IBL. Non-clonal algae were represented by 6 species. From each of these two groups we selected the 15 stands closer to the IBL (Fig. 5). The proximity of clonal algal stands to the IBL, when measured in ramet units, demonstrates that the general limitation of space occupation is of a purely physical nature: irrespective of non-clonal ramets competing for resources whereas in clonal ramets sharing resources there is a boundary that ramets are not able to cross. Norberg [54] proposed that the interspecific biomass–density boundary reflected a size-related design across species, and only the intraspecific self-thinning line reflected the biomass growth of individuals. However, the results also suggest that non-clonal algae are slightly more efficient than clonal ones when occupying space (Fig. 5). On average, the 15 non-clonal stands were 0.166 from the IBL whereas the 15 clonal stands were twice as distant (0.276). Permutation tests with 10,000 simulations determined that this difference was significant (*p* = 0.0001, *df*_within_ = 14, *df*_between_ = 1).

## Discussion

The position of the seaweed above the plant IBL can be interpreted in the light of classical models [1, 20] as a consequence of the aquatic habitat: frond size is limited by (i) volume available for occupation, in its turn set by the maximum possible frond height and the frond height-to-width ratio, both being structurally constrained, and (ii) biomass per unit volume, this being structurally as well as metabolically constrained.

Relative to point (i), the higher slope of the seaweeds’ IBL implies that they occupy more volume per unit surface [1, 20]. This may happen on account of seaweeds growing taller than plants when constrained to the same area. Recent plant models rely on structurally rigid tissue as determinant of the biomass–density relation [55] and on gravity as determinant of maximum plant height by imposing a limitation to the upward transport of water [56]. Seaweeds grow less constrained by these factors: the water column provides better support than air, reducing the need for very rigid tissues, and seaweeds are not limited by water nor need it for transpiration. Furthermore, when the larger seaweeds reach the top of the water column, they may bend and keep on elongating horizontally along the sea-surface [57]. Concomitantly, seaweeds may also occupy more volume per unit surface because they can keep their maximum width throughout their full heights (i.e., be cylindrically shaped). As they constantly move, seaweeds may also integrate the capture of light and nutrients resulting in a more homogeneous capture of resources throughout the canopy. Plants, on the other hand, have the shape limitations of their crowns disabling full use of the available volumes [58] and allowing light to pass by resulting in unused flecks on the forest floor; nutrient capture is also less homogeneous being dependent on the (fixed) position of roots. The comparison among experimentally determined slopes and the − 0.5 slope reflecting the theoretical full use of the available volume is enlightening: plants cannot achieve it (*β*_1_ = − 0.33) whereas seaweeds overcome it (*β*_1_ = − 0.67), implying that seaweeds partially overlap the volumes occupied by each individual within a fully crowded stand. To sustain this hypothesis we highlight that by being flexible neighbouring seaweeds are in constant motion, overlap their surface usage and move with the currents which adjust their position constantly. Finally, seaweeds are attached to the substrate by holdfasts that are proportionally smaller compared to roots, and hence are less likely to interfere with each other.

Relative to point (ii), although the higher intercept of seaweeds may result from growing relatively taller than plants (i.e., become slender)—an alternative way to use more volume per unit surface available—it may also result from packing more biomass per unit of used volume [1, 20], implying a more efficient use of nutrients and energy, and less competition. Again, the watery environment facilitates this aspect of seaweed life. Seaweeds acquire nutrients through, and photosynthesise over their entire surface, whereas plants mainly uptake nutrients and water through the roots, have non-areal (and often some areal) heterotrophic parts, and expend energy in aboveground structure and complex transport systems. Light scatters more in the sea than in the air. Therefore, seaweeds can receive more multidirectional light.

The contrast between plants and seaweeds described above is corroborated by the algal efficiency of space occupation decreasing with their structural complexity. Overall, the hypothesis follows: terrestrial plants have their efficiency of space occupation constrained by gravity, supply of water and nutrients, and the energetic requirements to transport these upwards [56, 59]. Overcoming these constrains demands structural and functional complexity, which in turn slows down growth. Inhabiting watery environments, seaweeds are relieved of the same constrains of terrestrial plants, and thus do not require the same structural and functional adaptations. Thus, the relationship is determined by the biomass packed into the volume effectively used, which seaweeds do most efficiently by tending to have simpler body constructions that also allow them to better accumulate biomass. Concomitantly with the benefit of simpler body constructions, the better efficiency of space occupation by non-clonal algae suggests that competition and elimination of the weaker ramets promotes better efficiency of space occupation than cooperation among ramets. Still, more species and stands of both types of algae, or manipulative experiments of clonal algae, are required for more conclusive results.

Many of the land plant stands with highest biomasses come from man-influenced or induced forestry systems which receive energy subsidies from human planting, managing, and caring activities that natural seaweed stands do not receive. But although constrained to their natural habitat, seaweeds such as the bull kelp (*Durvillaea antarctica*) still outperform plants. *D. antarctica* is a large, robust species with a circumpolar distribution which can dominate exposed rocky shores in southern New Zealand, Chile, parts of Argentina and the Kerguelen Islands. It lacks the air bladders common to most large-sized seaweeds, being held aloft to the light due to a unique honeycomb structure within the algae’s blades which also helps the kelp avoid being damaged by the strong waves [60]. It is nevertheless a simpler and less constraining solution suited to a less demanding environment, than is the overall rigid body construction taken by plants. The bull kelp often occurs where there is coastal nutrient upwelling and strong waves enhance mixing, thus increasing the replenishment of the nutrients that the *D. antarctica* can uptake through the whole of its surface. Being so abundant and edible, the biomass of Bull kelp is highly exploited for human food in Chile [35], which is not surprising as humans are optimal foragers which usually target the highest biomass edible food sources.

## Conclusions

Like plants, all algae (i.e., also clonal algae) are limited by their efficiency of space occupation. This limitation is of a purely physical nature and is superimposed on resource availability. Algae occupy space more efficiently than plants, most likely because the watery environment facilitates the physical process of space occupation. Furthermore, the efficiency of space occupation is a useful ecological indicator that can be used to discriminate among distinct algal groups.

## Additional files

**Additional file 1.** Table of sources of information and information extracted from sources. source, species name, number of observations of density and biomass, geographical location, latitude, phylum, taxa used, functional group data.

**Additional file 2.** Worldwide algal biomass–density data. Spreadsheet of all complied algae biomass and density data of each of the 56 studies on 42 algal species scattered worldwide and supplementary graphs used in the meta-analysis.

**Additional file 3.** Regression analysis software. Matlab software pack with the quantile regression used to estimate the IBL, as well as with alternative regression methods.

## Abbreviations

B: biomass
D: density
d_algae_: perpendicular distance to the algae IBL
IBL: interspecific boundary line
OLS: ordinary least squares
PCA: principal components analysis
QR: quantile regression
QR1: model 1 quantile regression
QR2: model 2 quantile regression
RMA: reduced major axis
w: individual plant weight

## References

[1] Yoda K, Kira T, Ogawa H, Hozumi K (1963). Self-thinning in overcrowded pure stands under cultivated and natural conditions (Intraspecific competition among higher plants. XI). J Biol.

[2] White J (1980). Demography and evolution in plant populations (ed O.T. Solbrig).

[3] Lonsdale WM, Watkinson AR (1983). Plant geometry and self-thinning. J Ecol.

[4] Weller DE (1987). A reevaluation of the − 3/2 power rule of plant self-thinning. Ecol Monogr.

[5] Hutchings MJ (1983). Ecology’s law in search of a theory. New Sci.

[6] White J, Harper JL (1970). Correlated change in plant size and number in plant populations. J Ecol.

[7] Hughes RN, Griffiths CL (1988). Self-thinning in barnacles and mussels: the geometry of packing. Am Nat.

[8] Rincón PA, Lbón-Cerviá J (2002). Nonlinear self-thinning in a stream-resident population of brown trout (*Salmo trutta*). Ecology.

[9] Gorham E (1979). Shoot height, weight and standing crop in relation to density of monospecific plant stands. Nature.

[10] Westoby M, Howell J (1986). Influence of population structure on self-thinning of plant populations. J Ecol.

[11] Westoby M (1981). The place of the self-thinning rule in population dynamics. Am Nat.

[12] Lonsdale WM (1990). The self-thinning rule: dead or alive?. Ecology.

[13] Weller DE (1991). The self-thinning rule: dead or unsupported?—a reply to Lonsdale. Ecology.

[14] Scrosati R (2005). Review of studies on biomass–density relationships (including self-thinning lines) in seaweeds: main contributions and persisting misconceptions. Phycol Res.

[15] Zhang L, Bi H, Gove JH, Heath LS (2005). A comparison of alternative methods for estimating the self-thinning boundary line. Can J For Res.

[16] Weller DE (1987). Self-thinning exponent correlated with allometric measures of plant geometry. Ecology.

[17] Morris EC, Myerscough PJ (1991). Self-thinning and competition intensity over a gradient of nutrient availability. J Ecol.

[18] Morris EC (2003). How does fertility of the substrate affect intraspecific competition? Evidence and synthesis from self-thinning. Ecol Res.

[19] Steen H, Scrosati R (2004). Intraspecific competition in *Fucus serratus* and *F. evanescens* (Phaeophyceae: Fucales) germlings: effects of settlement density, nutrient concentration, and temperature. Mar. Biol..

[20] Weller DE (1989). The interspecific size-density relationship among crowded plant stands and its implications for the − 3/2 power rule of self-thinning. Am Nat.

[21] Scrosati RA (2000). The interspecific biomass–density relationship for terrestrial plants: where do clonal red seaweeds stand and why?. Ecol Lett.

[22] Schiel DR, Choat JH (1980). Effects of density on monospecific stands of marine algae. Nature.

[23] Cousens R, Hutchings MJ (1983). The relationship between density and mean frond weight in monospecific seaweed stands. Nature.

[24] Robertson BL (1987). Reproductive ecology and canopystructure of *Fucus spiralis* L. Bot..

[25] Cheshire AC, Hallam ND (1988). Biomass and density of native stands of *DurvilIaea potatorum* (southern bull-kelp) in south eastern Australia. Mar Ecol Prog Ser.

[26] Russell G (1990). Age and stage in seaweed populations: a cautionary tale. Brit Phycol J.

[27] Martínez E, Santelices B (1992). Size hierarchy and the − 3/2 ‘power law’ relationship in a coalescent seaweed. J Phycol.

[28] Flores-Moya A, Fernández JA, Niell FX (1997). Growth pattern, reproduction, and self-thinning in seaweeds: a re-evaluation in reply to Scrosati. J Phycol.

[29] Scrosati R (1997). On the analysis of self-thinning among seaweeds. J Phycol.

[30] Creed JC (1995). Spatial dynamics of a *Himanthalia elongate* (Fucales, Phaeophyta) population. J Phycol.

[31] Creed JC, Kain JM, Norton TA (1998). An experimental evaluation of density and plant size in two large brown seaweeds. J Phycol.

[32] Arenas F, Fernández C (2000). Size structure and dynamics in a population of *Sargassum muticum* (Phaeophyceae). J Phycol.

[33] Haberl HK, Erb H, Krausmann F, Gaube V, Bondeau A, Plutzar C, Gingrich S, Lucht W, Fischer-Kowalski M (2007). Quantifying and mapping the human appropriation of net primary production in earth’s terrestrial ecosystems. Proc Natl Acad Sci USA.

[34] Lawton RJ, de Nys R, Paul NA (2013). Selecting reliable and robust freshwater macroalgae for biomass applications. PLoS ONE.

[35] Bustamante RH, Castilla JC (1990). Impact of human exploitation on populations of the intertidal southern bull-kelp *Durvillaea antarctica* (Phaeophyta, Durvilleales) in central Chile. Biol Cons.

[36] Hutchings MJ (1979). Weight–density relationships in ramet populations of clonal perennial herbs, with special reference to the 3/2 power law. J Ecol.

[37] Westoby M (1984). The self-thinning rule. Adv Ecol Res.

[38] de Kroon H, Kalliola R (1995). Shoot dynamics of the giant grass *Gynerium sagittatum* in Peruvian Amazon floodplains, a clonal plant that does show self-thinning. Oecologia.

[39] Lazo ML, Chapman ARO (1998). Components of crowding in a modular seaweed: sorting through the contradictions. Mar Ecol Prog Ser.

[40] Scrosati R, Servière-Zaragoza E (2000). Ramet dynamics for the clonal seaweed *Pterocladiella capillacea* (Rhodophyta): a comparison with *Chondrus crispus* and with *Mazzaella cornucopiae* (Gigartinales). J Phycol.

[41] Rivera M, Scrosati R (2008). Self-thinning and size inequality dynamics in a clonal seaweed (*Sargassum lapazeanum*, Phaeophyceae). J Phycol.

[42] Scrosati R (2006). Crowding in clonal seaweeds: does self-thinning occur in *Mastocarpus papillatus* shortly before stand biomass peaks?. Aquat Bot.

[43] Van Tussenbroek BI (1993). Plant and frond dynamics of the giant kelp, *Macrocystis pyrifera*, forming a fringing zone in the Falkland Islands. Eur J Phycol.

[44] Van Tussenbroek BI (1989). Seasonal growth and composition of fronds of *Macrocystis pyrifera* in the Falkland Islands. Mar Biol.

[45] Neushul M (1963). Studies on the giant kelp, *Macrocystis*. II. Reproduction. Am J Bot.

[46] Cormaci M, Furnari G, Scammacca B, Alongi G (1996). Summer biomass of a population of *Iridaea cordata* (Gigartinaceae, Rhodophyta) from Antartica. Hydrobiologia.

[47] Cormaci M, Furnari G, Scammacca B, Alongi G, Catra M (1998). Summer biomass of a population of *Phyllophora antartica* (Phyllophoraceae, Rhodophyta) from Antartica. Hydrobiologia.

[48] Goreau TJ, Trench RK (2013). Innovative methods of marine ecosystem restoration Boca Raton.

[49] Vieira VMNCS, Creed J, Scrosati RA, Santos A, Dutschke G, Leitão F (2016). On the choice of linear regression algorithms. Annu Res Rev Biol..

[50] Jackson JE (1991). A user’s guide to principal components.

[51] Draper NR. Straight line regression when both variables are subject to error. In: Proceedings of the 1991 Kansas State university conference on applied statistics in agriculture. 1992. p. 1–18.

[52] Smith RJ (2009). Use and misuse of reduced major axis for line-fitting. Am J Phys Anthropol.

[53] Steneck RS, Dethier MN (1994). A functional group approach to the structure of algal-dominated communities. Oikos.

[54] Norberg RA (1988). Theory of growth geometry of plants and self-thinning of plant populations: geometric similarity, elastic similarity, and different growth modes of plant parts. Am Nat.

[55] Franco M, Kelly CK (1998). The interspecific mass–density relationship and plant geometry. Proc Natl Acad Sci.

[56] Koch GW, Sillett SC, Jennings JM, Davis SD (2004). The limits to tree height. Nature.

[57] Reed D, Rassweiller A, Arkema K (2009). Density derived estimates of standing crop and net primary production in the giant kelp *Macrocystis pyrifera*. Mar Biol.

[58] Purves DW, Lichstein JW, Pacala SW (2007). Crown plasticity and competition for canopy space: a new spatially implicit model parameterized for 250 North American tree species. PLoS ONE.

[59] Álvarez-Dávila E, Cayuela L, González-Caro S, Aldana AM, Stevenson PR, Phillips O (2017). Forest biomass density across large climate gradients in northern South America is related to water availability but not with temperature. PLoS ONE.

[60] Fraser CI, Hay CH, Spencer HG, Waters JM (2009). Genetic and morphological analyses of the southern Bull Kelp Durvillaea antarctica (Phaeophyceae: Durvillaeales) in New Zealand reveal cryptic species. J Phycol.

