# Supplementary material for: A meta-analysis shows that seaweeds surpass plants, setting life-on-Earth’s limit for biomass packing
Source: BMC Ecol. 2019 Jan 31;19:6. doi: 10.1186/s12898-019-0218-z (PMC6357480; doi:10.1186/s12898-019-0218-z)
Supplement: Supplementary file 1 — Additional file 1. Table of sources of information and information extracted from sources. source, species name, number of observations of density and biomass, geographical location, latitude, phylum, taxa used, functional group data. [file 12898_2019_218_MOESM1_ESM.docx]

Additional Table 1: Sources of information and information extracted from sources: source, species name, number of observations of density and biomass, geographical location, latitude, phylum, taxa used, functional group.

| Species | Source | nº obs. | Location | Latitude | Phylum | Taxa used | Functional group |
| --- | --- | --- | --- | --- | --- | --- | --- |
| *Alaria esculenta* ^(n)^ | Merzouka and Johnson (2011) | 1 | Northwest Canada | 48.45 | *Ochrophyta* | *Laminariales* | Leathery macrophyte |
| *Alaria esculenta* ^(n)^ | Creed unpublished | 24 | Port Erin, Isle of Man, UK | 54.008 | *Ochrophyta* | *Laminariales* | Leathery macrophyte |
| *Ascophyllum nodosum* ^(c)^ | Golletya et al. (2011) | 5 | Britany, France | 48 | *Ochrophyta* | *Fucales* | Leathery macrophyte |
| *Ascophyllum nodosum* ^(c)^ | Cousens and Hutchings (1983) | 5 | n.d. | n.d. | *Ochrophyta* | *Fucales* | Leathery macrophyte |
| *Asparagopsis armata* ^(c)^ | Flores-Moya et al (1997) | 48 | Tarifa, Strait of Gibraltar, Spain | 36 | *Rhodophyta* | *Bonnemaisoniales* | Corticated macrophyte |
| *Chondrus crispus* ^(c)^ | Chopin et al. (1992) | 15 | Prince Eduard Island, Canada | 46.6 | *Rhodophyta* | *Gigartinales* | Corticated macrophyte |
| *Chordaria flagelliformis* ^(n)^ | Rice and Chapman (1982) | 11 | Coote Cove; Cranberry Cove, Nova Scotia, Canada | 44.47 | *Ochrophyta* | *Ectocarpales* | Corticated macrophyte |
| *Codium bursa* ^(n)^ | Vidondo and Duarte (1998). | 10 | Cala Jouquet, Spain | 42.3 | *Chlorophyta* | *Chlorophyta* | Corticated macrophyte |
| *Desmarestia firma* ^(n)^ | Anderson and Hay (1986) | 11 | Luderitz Bay and Cape Point, South Africa | -29 | *Ochrophyta* | *Desmasrestiales* | Corticated macrophyte |
| *Durvillaea Antarctica* ^(n)^ | Lawrence (1986) | 24 | Kerguelen, France | 49 | *Ochrophyta* | *Fucales* | Leathery macrophyte |
| *Durvillaea potatorum* ^(n)^ | Cheshire and Hallam (1988) | 31 | Southeast Australia | -40 | *Ochrophyta* | *Fucales* | Leathery macrophyte |
| *Ecklonia cava*^(n)^ | Watanuki and Yamamoto (1990) | 21 | Sagami Bay, Japan | 33 | *Ochrophyta* | *Laminariales* | Leathery macrophyte |
| *Ecklonia radiata* ^(n)^ | Kirkman (1985) | 22 | Marmion Reef, Whitfords, Australia | -31.8 | *Ochrophyta* | *Laminariales* | Leathery macrophyte |
| *Ecklonia radiata* ^(n)^ | Schiel and Choat. (1980) | 7 | Northern New Zealand | n.d. | *Ochrophyta* | *Laminariales* | Leathery macrophyte |
| *Enteromorpha intestinalis* ^(n)^ | Creed unpublished | 1 | Port. St. Mary, Isle of Man, UK | 54.066 | *Chlorophyta* | *Chlorophyta* | Foliose algae |
| *Fucus distichus* ^(n)^ | Thom (1983) | 24 | Puget Sound, USA | n.d. | *Ochrophyta* | *Fucales* | Leathery macrophyte |
| *Fucus evanescens* ^(n)^ | Steen and Scrosati (2004) | 44 | Langesund, Skagerrak Strait, Norway | 58.98 | *Ochrophyta* | *Fucales* | Leathery macrophyte |
| *Fucus serratus* ^(n)^ | Steen and Scrosati (2004) | 45 | Langesund, Skagerrak Strait, Norway | 58.98 | *Ochrophyta* | *Fucales* | Leathery macrophyte |
| *Fucus serratus* ^(n)^ | Karez (2003) | 28 | Helgoland Island, Germany | 54.18 | *Ochrophyta* | *Fucales* | Leathery macrophyte |
| *Fucus serratus* ^(n)^ | Creed et al. (1998) | 112 | Port Erin, Isle of Man, UK | 54.08 | *Ochrophyta* | *Fucales* | Leathery macrophyte |
| *Fucus serratus* ^(n)^ | Creed unpublished | 12 | Poyll Richie, Isle of Man, UK | 54.066 | *Ochrophyta* | *Fucales* | Leathery macrophyte |
| *Fucus spiralis* ^(n)^ | Karez (2003) | 25 | Helgoland Island, Germany | 54.18 | *Ochrophyta* | *Fucales* | Leathery macrophyte |
| *Fucus spiralis* ^(n)^ | Robertson (1987) | 24 | Ketch Harbour, Canada | 44.48 | *Ochrophyta* | *Fucales* | Leathery macrophyte |
| *Fucus vesiculosus* ^(n)^ | Karez (2003) | 30 | Helgoland Island, Germany | 54.18 | *Ochrophyta* | *Fucales* | Leathery macrophyte |
| *Fucus vesiculosus* ^(n)^ | Creed unpublished | 10 | Port. St. Mary, Isle of Man, UK | 54.066 | *Ochrophyta* | *Fucales* | Leathery macrophyte |
| *Fucus vesiculosus* ^(n)^ | Creed (1993) (Thesis) | 84 | Port. St. Mary, Isle of Man, UK | 54.066 | *Ochrophyta* | *Fucales* | Leathery macrophyte |
| *Gelidium sesquipedale* ^(c)^ | Santos (1995) | 51 | Cape Espichel, Portugal | 38.4 | *Rhodophyta* | *Gelidiales* | Corticated macrophyte |
| *Himanthalia elongata* ^(n)^ | Russel (1990) | 71 | Port St. Mary, Isle of Man, UK | 54.066 | *Ochrophyta* | *Fucales* | Leathery macrophyte |
| *Himanthalia elongata*^(n)^ | Creed (1995) | 30 | Port. St. Mary, Isle of Man, UK | 54.066 | *Ochrophyta* | *Fucales* | Leathery macrophyte |
| *Himanthalia elongata* ^(n)^ | Creed (1993) (Thesis) | 112 | Port. St. Mary, Isle of Man, UK | 54.066 | *Ochrophyta* | *Fucales* | Leathery macrophyte |
| *Himanthalia elongata* ^(n)^ | Creed unpublished | 3 | Port. St. Mary, Isle of Man, UK | 54.066 | *Ochrophyta* | *Fucales* | Leathery macrophyte |
| *Hormosira banksii* ^(n)^ | Schiel and Lilley (2011) | 5 | Kaikoura and Moeraki, New Zealand | -43.875 | *Ochrophyta* | *Fucales* | Leathery macrophyte |
| *Iridaea cordata* ^(c)^ | Cormaci *et al*. (1996) | 5 | Terra Nova Bay, Ross Sea, Antartica | -74.7 | *Rhodophyta* | *Gigartinales* |  |
| *Laminaria digitata*^(n)^ | Smith (1986) | 5 | Ram Island, Lobster Bay, New Scotia, Canada | 43.68 | *Ochrophyta* | *Laminariales* | Leathery macrophyte |
| *Laminaria digitata*^(n)^ | Creed et al (1998) | 301 | Port. Erin, Isle of Man, UK | 54.08 | *Ochrophyta* | *Laminariales* | Leathery macrophyte |
| *Laminaria digitata*^(n)^ | Creed unpublished | 8 | Port. St. Mary, Isle of Man, UK | 54.066 | *Ochrophyta* | *Laminariales* | Leathery macrophyte |
| *Laminaria hyperborea* ^(n)^ | Jupp and Drew (1974) | 10 | Arisaig, Invernesshire, Scotland | 56.95 | *Ochrophyta* | *Laminariales* | Leathery macrophyte |
| *Laminaria longicruris* ^(n)^ | Smith (1986) | 5 | Ram Island, Lobster Bay, New Scotia, Canada | 43.68 | *Ochrophyta* | *Laminariales* | Leathery macrophyte |
| *Laminaria longicruris* ^(n)^ | Gerard and Mann (1979) | 2 | Shag Bay, New Scotia, Canada | 43.48 | *Ochrophyta* | *Laminariales* | Leathery macrophyte |
| *Laminaria saccharina* ^(n)^ | Creed unpublished | 3 | Port. St. Mary, Isle of Man, UK | 54.066 | *Ochrophyta* | *Laminariales* | Leathery macrophyte |
| *Leathesia difformis* ^(n)^ | Chapman and Goudey (1983) | 4 | n.d. | n.d. | *Ochrophyta* | *Ectocarpales* | Corticated foliose |
| *Macrocystis pyrifera* ^(n)^ | Reed et al. (2009) | 19 | Santa Barbara, California, USA | 34.4 | *Ochrophyta* | *Laminariales* | Leathery macrophyte |
| *Macrocystis pyrifera* ^(n)^ | Tussenbroek (1993) | 2 | Falkland Islands, United Kingdom | -52 | *Ochrophyta* | *Laminariales* | Leathery macrophyte |
| *Mastocarpus papillatus* ^(c)^ | Scrosati (2006) | 4 | Acadia Beach, Vancouver, Canada. | 49.28 | *Rhodophyta* | *Gigartinales* | Corticated foliose |
| *Mazzaella cornucopiae* ^(c)^ | Scrosati and De Wreede (1997) | 98 | Prasiola Point, Vancouver Is., Canada | 48.816 | *Rhodophyta* | *Gigartinales* | Corticated foliose |
| *Mazzaella cornucopiae* ^(c)^ | Scrosati and De Wreede (1997) | 12 | Prasiola Point, Vancouver Is., Canada | 48.816 | *Rhodophyta* | *Gigartinales* | Corticated foliose |
| *Phyllariopsis purpurascens* ^(n)^ | Flores-Moya et al. (1997) | 6 | Tarifa, Strait of Gibraltar, Spain | 36 | *Ochrophyta* | *Tilopteridales* | Leathery macrophyte |
| *Phyllophora antartica* ^(c)^ | Cormaci *et al*. (1998) | 5 | Terra Nova Bay, Ross Sea, Antartica | -74.7 | *Rhodophyta* | *Gigartinales* |  |
| *Pterocladiella capillacea* ^(c)^ | Scrosati and Servière-Zaragoza (2000) | 56 | Lobos Point, Baja California Sur, Mexico | 23.416 | *Rhodophyta* | *Gelidiales* | Corticated foliose |
| *Pterocladiella capillacea* ^(c)^ | Scrosati (2000) | 20 | Lobos Point, Baja California Sur, Mexico | 23.416 | *Rhodophyta* | *Gelidiales* | Corticated foliose |
| *Saccorhiza polyschides* ^(n)^ | Cousens and Hutchings (1983) | 6 | Asturias, Spain | 43.58 | *Ochrophyta* | *Fucales* | Leathery macrophyte |
| *Sargassum ilicifolium*^(c)^ | Ateweberhan et al. (2009) | 42 | Massawa, Red Sea, Eritreia | 15.59 | *Ochrophyta* | *Fucales* | Leathery macrophyte |
| *Sargassum lapazeanum* ^(c)^ | Rivera and Scrosati (2008) | 6 | La Paz, Baja California Sur, Mexico | 24.35 | *Ochrophyta* | *Fucales* | Leathery macrophyte |
| *Sargassum muticum*^(c)^ | Baer and Stengel (2010) | 20 | Ireland | 53.35 | *Ochrophyta* | *Fucales* | Leathery macrophyte |
| *Sargassum muticum*^(c)^ | Arenas and Fernández (2000) | 41 | Cape Peñas, Asturias, Spain | 43.6 | *Ochrophyta* | *Fucales* | Leathery macrophyte |
| *Sargassum muticum*^(c)^ | Andrew and Viejo (1998) | 84 | Aramar, Spain | 43.6 | *Ochrophyta* | *Fucales* | Leathery macrophyte |
| *Sargassum polyceratium*^(c)^ | Engelen et al. (2005) | 3 | Curaçao, Dutch Antilles | 12 | *Ochrophyta* | *Fucales* | Leathery macrophyte |
| *Sargassum sinclairii*^(c)^ | Schiel and Choat (1980) | 9 | Northern New Zealand | n.d. | *Ochrophyta* | *Fucales* | Leathery macrophyte |
| *Sargassum subrepandum*^(c)^ | Ateweberhan et al. (2009) | 30 | Massawa, Red Sea, Eritreia | 15.59 | *Ochrophyta* | *Fucales* | Leathery macrophyte |
| *Turbinaria triquetra* ^(c)^ | Ateweberhan et al. (2009) | 63 | Massawa, Red Sea, Eritreia | 15.59 | *Ochrophyta* | *Fucales* | Leathery macrophyte |
| *Turbinaria triquetra* ^(c)^ | Ateweberhan et al. (2009) | 38 | Massawa, Red Sea, Eritreia | 15.59 | *Ochrophyta* | *Fucales* | Leathery macrophyte |
