# Supplementary material for: A meta-analysis shows that seaweeds surpass plants, setting life-on-Earth’s limit for biomass packing
Source: BMC Ecol. 2019 Jan 31;19:6. doi: 10.1186/s12898-019-0218-z (PMC6357480; doi:10.1186/s12898-019-0218-z)
Supplement: Supplementary file 3 — Additional file 3. Regression analysis software. Matlab software pack with the quantile regression used to estimate the IBL, as well as with alternative regression methods. [file 12898_2019_218_MOESM3_ESM.zip › software ANCOVA/Tutorial.doc]

**1.	Overview**

This Matlab-based software implements non-parametric tests to several features of regression analysis and ANCOVA designs. It is user friendly, demanding little experience in programming and in Matlab, and has built-in options to import data from Excel. Its first stages are dedicated to the slopes. Their parent file is the SlopesExe.m, invoking the child file SlopeTest.m to assess the significance of each slope, and the child file SlopeDiffTest.m to assess the significance of differences between pair-wised slopes. If the data corresponds to time-series with replicates, the non-parametric algorithm is adapted to randomly permute within each time instance, which provides better results. Following the ANCOVA design, when slopes are significant but not significantly different among them, a posterior stage is dedicated to the significance of difference between pair-wised intercepts. Its parent file is the InterceptExe.m, invoking the child file InterceptTest.m. The results are provided in data structures containing the respective metrics and their significances. The regressions are automatically plotted.

**1.	Slope test overview**

In the first sections of the parent file SlopesExe.m the user edits the analysis choosing (i) how to import data, (ii) how to pre-process data, (iii) the type of regression method, (iv) test significances of slopes, and (v) test significances of differences between slopes. The latter sections of the parent file SlopesExe.m control the calculus and are not to be edit by the user.

**1.1.	Import data**

The data can be imported from Excel files or already be in Matlab format (.mat files). This is done in line 4.

If the data is in an Excel file the software imports it into the Matlab workspace using the xlsread function. Then, it is required to perform the following edits:

(i)	the directory with the excel file is defined in line 9.

(ii) the name of the file is defined in line 13.

(iii) the name of the worksheet within the file is defined in line 17.

(iv) the data range with the x, y and categorical variable(s) is defined in line 23 and stored in the m×n cell array named RawData. The m is the number of observations while the n is the c+2 number of variables including the 2 numerical plus the c categorical. The numeric variables are automatically converted to a numeric array named Data while the categorical variable(s) is automatically stored in the cell array observations.

If the data is in Matlab format the respective .mat file must be loaded in advance to the Matlab workspace. Then, it is required to perform the following edits:

(i)	the numeric data array is defined in line 48. This is a m×2 matrix with x in the first m×1 column vector and y in the second. The m is the number of available observations. Yet, not all must be used, in which case it is required to provide an array identifying the observations to use. See the examples below.

(ii) the names of the x and y numeric variables are stored in the 2×1 cell array as defined in line 51.

(iii)	the categorical data array i.e., the names of each x and y observation are stored in the m×1 cell array. This is done in line 54. Several observations can (and should) have the same name, indicating that these belong to the same group. Hence, all observations may be discriminated among groups.

The names of the groups identifying and differentiating the observations are defined in line 61.

**1.2.	Pre-process data**

The data pre-processing involves three stages. In the first stage the observations are discriminated according to their group. As said above, these groups are defined in line 61. The second stage removes (or not) the outliers (in Line 95) and depending on its type [1,2] (in Line 98). The outliers specific to a variable likely related to measurement error. They are identified in the plots by a blue triangle. The eigenvector’s outliers are common to both variables and likely report the occurrence of an extreme event. The first eigenvector’s outliers are identified by a red circle and the second eigenvector’s by a red square. Then, there is the threshold to considered the observation an outlier. The probability of finding values on the tails of the distribution depends on the sample size. In samples sized several hundreds or thousands it is practically inevitable the occurrence of observations in the p<0.01 tail of the distribution. Thus, in Line 101 this threshold is defined as a fraction (p_out_) of the proportion of each observation within the sample (1/n) i.e., threshold = p_out_ ×1/n. In the third stage the observations are standardized to their group’s mean.

**1.3.	Regression method**

Among the options provided in this software, the most commonly used by the research community is Ordinary Least Squares (OLS). It belongs to the Model I Regression class, regressing the y dependent variable (response) into the x independent variable (predictor) by minimizing the sum of squares of the vertical residuals. In a set of situations it is more adequate to simultaneously regress y into x and vice-versa. This is done by Model II Regression minimizing the residuals obliquely in the x-y plan under the restriction of being perpendicular to the obtained regression line [3-8]. The software includes the two most common methods of this class, namely: Principal Components Analysis (PCA) and Reduced Major Axis (RMA). It also includes the intricate sub-class of Errors-in-the-Variables with several options for the Method of the Moments (the M acronyms) and the Method of the Cumulants (the K acronyms) [9-12]. All methods above estimate the average trend. Alternatively, the Quantile Regression (QR) estimates the τ quantile of the trend. The software provides as optional Model I and Model II Quantile Regression. In line 77 is chosen the regression type. In line 81 is chosen the Variance-Covariance Matrix (specific for algorithms stabilizing the Methods of the Moments). In line 85 is chosen optimization algorithm (specific for algorithms stabilizing the Methods of the Moments). In line 88 is defined the expected error in x estimates (specific for algorithms stabilizing the Methods of the Moments). In line 91 is defined the τ quantile to estimate the QR coefficients. In line 92 is chosen the increment in x and y used to estimate numerical derivatives for the linear programming algorithm.

**1.4.	Significance of slopes**

In line 64 it is chosen whether to test the significance of each slope. This is done by permutation tests, which simulate the null hypothesis by randomly redistributing both variables independently from each other, thus breaking their correlation. In this case, the correlation coefficient and slope obtained are the result of random error. Randomly redistributing the empirical data many times provides the empirical distribution of the correlation coefficient and of the slope under the null hypothesis [13-16].

**1.5.	Significance of differences between slopes**

the significance of differences between slopes is assessed pair-wised by the child file SlopeDiffTest.m. selecting in line 107 one from several available methods, namely:

(i)	permutation tests simulate the null hypothesis that both slopes come from the same statistical population by randomly redistributing the observations among groups. Yet, this requires standardizing the observations within each group to have zero mean. This test was proved the more robust [15,16].

(ii)	the overlap of the bootstrapped confidence intervals. The decision of whether the slopes are significantly different depends on whether they overlap their confidence intervals estimated by bootstrap [17] for a percentile defined in line 116. In this case, there is not a null hypothesis and so, it is not a true test of significance [15,16].

(iii)	the t-student is the standard parametric test.

The number of randomizations to perform, either in the permutation tests or in the bootstrap tests, is defined in line 113. If the data corresponds to time-series with replicates (defined in line 110), an adaptation implemented by SlopeDiffTestRTS.m provides better results [16]. In this case it must be defined:

(i)	the names of the replicates (in line 124) may be numeric (provided in a double array) or alpha-numeric (provided in a cell array). These names must be common for all groups e.g., if one group has replicates R1 to R4, all other groups must follow this labelling starting from R1 to Rn.

(ii)	the array with the names of the replicates (line 127).

(iii)	the time instances when the replicates where sampled (in line 130) must be numeric (preferably) integers sorted ascendingly (preferably starting from 1). Each replicate need not have been sampled at every time instance. But the names of the time instances must be common for all replicates.

(iv) the array with the names of the replicates (line 133).

(v)	whether the observations are standardized within groups or within replicates (line 136).

(vi)	whether to plot the standardized timeseries (line 139).

(vii)	and the colour to plot each replicate (line 142).

**2.1.	Significance of differences between intercepts**

Line 85: choose whether to test the significance of the differences between intercepts.

**3.	Examples**

Example 1: the dados.mat file with the algal biomass-density data was loaded into the workspace. The numeric array was built horizontally concatenating both 1856×1 column-vectors with the log_10_ densities and log_10_ biomasses. Hence, line 48 was Data = [log10D log10B];

The variables’ names are store in the 2×1 cell array, defined in line 51 as variables = {‘log10D’ ‘log10B’};

We wished to estimate the central tendency of each red algae order and compare among them, leaving the brown algae and green algae out. In line 54, the cell array was defined as observations = Taxa;

In line 61 was defined the cell array with the names of each observed order intended to use in the analysis.

groups = {‘Gelidiales’ ‘Gigartinales’ ‘Bonnemaisoniales’};

The software will select from the 1856 lines only the ones matching these names. Then, in line 64 we choose that we wish to test the significance of each slope and in line 67 we choose that we wish to test the significance of differences between slopes. In line 77 we choose to perform the Principal Components Analysis (PCA). In line 95 we choose to find the outliers. In line 98 we choose to remove only outliers in a specific variable as these most likely relate to measurements error. On the contrary, eigenvector outliers most likely relate to extreme biomass-density events, which are nevertheless valid and relevant for our analysis. In line 101 we set the outlier threshold for 1/5 of the proportion of each observation within its group. In line 107 we choose to test the differences between slopes using permutation tests. In line 110 we say that the data does not correspond to time-series. In line 113 we choose to tests both significances (for the slopes and for the differences between slopes) with 1000 randomizations.

Example 2: the data was previously imported in Matlab format, all the numeric data stored in the 1856×50 matrix, with the log10 density in column 18 and the log10 biomass in column 19. We wished to estimate the algal IBL from the 500 observations closer to the top-right corner of the log10B-log10D plot. These observations were previously determined and their row numbers identified in the 500×1 numeric array named ‘j500’. Consequently, in line 48, the numeric array was defined as Data = num(j500,18:19);

The column-vector with the observations’ names was previously defined as the 1856×1 cell array named all and with the word all also in each cell. In line 54, the cell array was defined as observations = all(j500);

In line 61 was defined the cell array with the name common to all observations: groups = {‘all’};

Hence, the software will select all the 500 observations (one observation per line) as all match this name. Then, in line 64 we choose that we wish to test the significance of this slope and in line 67 we choose that we do not wish to test the significance of differences between slopes (because there is only one slope). In line 77 we choose to perform the Model II Quantile Regression (QR2). This method requires specific settings, namely: in line 91 we choose the 0.99 quantile and in line 92 we set the optimization algorithm to search for the solution (i.e., the roots of the numerical partial derivatives) at intervals of ∆x= ∆y=0.02. In line 95 we choose not to find outliers and in line 98 we choose not to remove any outlier, as we had previously decided to work specifically with the top 500 observations. In line 113 we choose to test the significance of the slope with 10000 randomizations.

**3.	Refferences**

1.	Jackson JE. A user’s guide to principal components. John Wiley & Sons, New York, USA; 1991.

2.	Manly BJF. Multivariate methods. Chapman & Hall, London, UK; 1986.

3.	Pearson K. On lines and planes of closest fit to systems of points in space. Philos Mag. 1901;2:559-572.

4.	Ricker WE. Linear regressions in fishery research. J Fish Res Board Can. 1973;30: 409–434.

5.	Draper NR. Straight line regression when both variables are subject to error. In Proocedings of the 1991 Kansas State University Conference on Applied Statistics in Agriculture. 1992;1-18.

6.	Claude J. Morphometrics with R. Springer, New York, USA; 2008.

7.	Smith RJ. Use and misuse of Reduced Major Axis for line-fitting. Am J Phys Anthropol. 2009;140:476-486.

8.	Legendre P. Model II Regression User Guide, R Edition. 2013.

9.	Cragg JG. Using higher moments to estimate the simple errors-in-variables model. RAND J Econ. 1997;28(0):71-91.

10.	Dagenais MG, Dagenais DL. Higher moment estimators for linear regression models with errors in the variables. J Econometrics. 1997;76:193-221.

11.	Gillard JW, Iles TC. Method of moments estimation in linear regression with errors in both variables. Cardiff University School of Mathematics Technical Paper, Cardiff, UK; 2015.

12.	Gillard J. An overview of linear structural models in errors in variables regression. REVSTAT Stat J. 2010;8(1):57-80.

13.	Manly BJF. Randomization and Monte Carlo Methods in Biology. Chapman & Hall, London, UK; 1991.

14.	Dijksterhuis GB, Heiser WJ. The role of permutation tests in exploratory multivariate data analysis.
Food Quality and Preference, 1995;6: 263-270

15.	Vieira VMNCS, Creed J. Estimating significances of differences between slopes: A new methodology and software. Computational Ecology and Software. 2013;3(3):44-52.

16.	Vieira VMNCS, Creed J. Significances of differences between slopes: An upgrade for replicated time series. Computational Ecology and Software. 2013;3(4):102-109.

17.	Sokal RR, Rohlf FJ. Biometry: The principles and practice of statistics in biological research, 2nd ed. W.H. Freeman and company. New York, USA; 1981.

19.	Creasy MA. Confidence limits for the gradient in the linear functional relationship. J. Roy. Statist. Soc. Ser. B.
1956;18:65–69.

18.	Vieira VMNCS, Leitão Fand Mateus M. Biomass-density data analysis: A comment on Cabaço et al. (2013). J Ecol. 2015;103: 537-540. DOI: 10.1111/1365-2745.12294
